# RP-HPLC and Spectrophotometric Estimation of Ambroxol Hydrochloride and Cetirizine Hydrochloride in Combined Dosage Form

**DOI:** 10.4103/0250-474X.45398

**Published:** 2008

**Authors:** Neela M. Bhatia, S. K. Ganbavale, M. S. Bhatia, H. N. More, S. U. Kokil

**Affiliations:** Department of Pharmaceutical Chemistry, Bharati Vidyapeeth College of Pharmacy, Near Chitranagri, Kolhapur-416 013, India

**Keywords:** Ambroxol hydrochloride, cetirizine hydrochloride, RP-HPLC, absorbance ratio method

## Abstract

Rapid, precise, accurate, specific and sensitive reverse phase liquid chromatographic and absorbance ratio spectrophotometric methods have been developed for the simultaneous analysis of ambroxol hydrochloride and cetirizine hydrochloride in their tablet formulation. The chromatographic methods were standardized using a HIQ SIL-C_18_ column (250×4.6 mm i.d., 10 μm particle size) with UV detection at 229 nm and mobile phase consisting of methanol-acetonitrile-water (40:40:20, v/v/v). Ambroxol hydrochloride and cetirizine hydrochloride have absorbance maxima at 243 nm and 229 nm, respectively. The isoabsorptive wavelength for both the drugs was 236 nm. For absorbance ratio method developed, wavelengths selected were 243 nm and 236 nm. The proposed methods were successfully applied to the determination of ambroxol hydrochloride and cetirizine hydrochloride in tablets, with high percentage of recovery, good accuracy and acceptable precision. Different analytical performance parameters such as linearity, precision, accuracy, limit of detection, limit of quantitation and robustness were determined according to International Conference on Harmonization ICH Q2B guidelines. Results of analysis of the developed method were compared by performing ANOVA.

Ambroxol hydrochloride (AM) [trans-4-(2-amino-3,5-dibromobenzylamino) cyclohexanol Hydrochloride]^1^ is semi-synthetic derivative of vasicine obtained from Indian shrub *Adhatoda vasica.* It is a metabolic product of bromhexine. It is used as broncho secretolytic and expectorant drug^2^. It stimulates the transportation of the viscous secretions in the respiratory organs and reduces the stand stillness of the secretions. Several spectrophotometric methods have been reported for the qualitative and quantitative determination of AM from pharmaceutical formulations^3^–^6^. Various HPLC^7^–^10^, GLC^11^,^12^, LC-MS^13^ and Capillary electrophoretic^14^ methods are also reported for it's determination from biological fluids. Cetirizine hydrocloride (CE) [2-[4-(4-chlorobenzhydryl) piperazine-1-yl]ethoxyacetic acid] is the carboxylated metabolite of hydroxyzine and having high specific affinity for histamine H_1_ receptor. It is second generation antihistaminic drug. Literature survey revels that several spectrophotometric^15^–^18^ methods, HPLC methods^19^–^22^, HPLC coupled to tandem mass spectroscopy^23^, capillary electrophoretic^24^–^25^ have been also reported for determination of CE from pharmaceutical formulations and biological fluids.

The present research work describes rapid, accurate, sensitive and reproducible RP-HPLC method and absorbance ratio method for simultaneous determination of AM and CE from the tablet formulation.

## MATERIALS AND METHODS

AM was procured from Litaka Pharmaceuticals, Pune (India) and CE was kindly supplied as gift sample by Mediorals, Satara (India). Hydrochlorothiazide (HT) was supplied by (IPCA) Pharmaceuticals (India). All the reagents used were of analytical reagent grade. A commercial preparation (Relent tablets, Dr. Reddy's laboratories, India, Batch No: 2073) used for analysis was procured from the local pharmacy. Each film coated tablet contains 60 mg of AM and 5 mg of CE.

### HPLC method (method I):

HPLC Model–Jasco PU-2080 equipped with UV/Vis detector (Jasco UV–2075 plus), Column dimensions, HIQ SIL-C_18_ -column (250×4.6 mm i.d., 10 μm particle size)) Kya Tech, Japan with an isocratic mode was used at a flow rate of 1.0 ml/min at room temperature with injection volume of 20 μl and wavelength detection at 229 nm). The mobile phase used for RP-HPLC was methanol-acetonitrile-water (40:40:20, v/v/v).

### Preparation of standard stock solutions:

Standard stock solution containing AM and CE was prepared by dissolving 50 mg of AM and 5 mg CE in 40 ml of methanol. It was then sonicated for 10 min and then final volume of the solution was made up to 50 ml with methanol to get stock solution containing 1000 μg/ml of AM and 100 μg/ml of CE in 50 ml volumetric flask. Standard stock solution of HT was prepared by dissolving 5 mg of HT in 40 ml of methanol and then sonicated for 10 min. The final volume of solution was made up to 50 ml with methanol to get 100 μg/ml of HT.

### Linearity study:

**Fig. 1:**
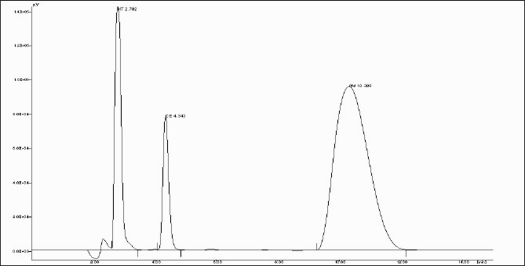
A typical chromatogram of HT, CE and AM A typical chromatogram of hydrochlorothiazide (HT), cetirizine hydrochloride (CE) and ambroxol hydrochloride (AM)

**Fig. 2:**
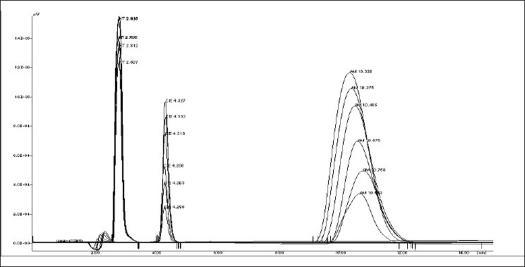
Overlain chromatograms of HT, CE and AM Overlain chromatograms of hydrochlorothiazide (HT), cetirizine hydrochloride (CE) and ambroxol hydrochloride (AM).

In to a series of 10 ml volumetric flasks, 1.2 to 4.8 ml of AM and 1 to 3.5 ml of CE solution were pipetted and to each flask 0.2 ml of HT was added and then final volume of the solutions was made up to 10 ml with methanol. A 20 μl of sample solution was injected into the injection port of chromatographic system having fixed volume loop injector. Chromatograms were noted and response factor was plotted against concentration to get calibration curve. Chromatogram of physical mixture of AM and CE is shown in fig. 1. Overlain chromatogram of physical mixture is shown in fig. 2. The regression equation data for the calibration curve is represented in Table 1 for AM and CE.

**TABLE 1:** METHOD VALIDATION PARAMETERS

Parameters	Method 1		Method 2	
	AM	CE	AM	CE	
Linearity range (μg/ml)	120-420	10-35	5-50	7.5-75
Correlation coefficient	0.9988	0.9993	--	--
Regression equation (Y*)				
Slope (B)	0.0235	0.0671	--	--
Intercept (A)	0.0098	0.0152	--	--
Precision (%CV)				
Repeatability of sample application n=9	0.83	0.89	0.92	1.04
Limit of Detection (LOD)**	0.0328	0.0648	1.12	0.45
Limit of Quantisation (LOQ)**	0.1076	0.2160	3.75	1.45
Specificity	Specific	Specific	Specific	Specific

* Y= A+B*C, where C is the concentration in μg/ml and Y is absorbance unit.

** Data obtained by nine determinations.

### Sample preparation:

From the triturate of 20 tablets, an amount equivalent to 30 mg of AM and 2.5 mg of CE was weighed and dissolved in 40 ml of methanol by sonicating for 10 min. The solution was filtered through 0.22 μ membrane filter and then final volume of the solution was made up to 100 ml with methanol to get stock solution containing 300 μg/ml of AM and 25 μg/ml of CE in 100 ml volumetric flask. After appropriate dilutions, the solutions were run on HPLC system and the concentration of each analyte was determined with the equations generated. The statistical data obtained after replicate determinations is shown in Table 2.

**TABLE 2:** RESULTS OF ANALYSIS

Method	Sample	Label claim (mg/tablet)	Concentration estimated*	%concentration estimated*	%RSD
Method I	Laboratory samples	AM 60	60.31	100.52	1.02
		CE5	4.96	99.27	0.78
	Tablets	AM 60	60.14	100.24	0.86
		CE5	5.02	100.43	0.97
Method II	Laboratory samples	AM 60	60.68	101.14	0.62
		CE5	4.96	99.29	1.11
	Tablets	AM 60	60.43	100.73	0.67
		CE5	5.06	101.23	0.92

* Average of nine determinations; %RSD = Relative standard deviation

### Absorbance ratio method (Method II)

A PC based Jasco V-530 recording spectrophotometer with spectral bandwidth of 2 nm and wavelength accuracy±0.5 nm (with automatic wavelength correction) was employed for all measurements using a matched pair of 10 mm quartz cell. Shimadzu AY 120 analytical balance was used for weighing. Methanol HPLC grade was purchased from Merck Pharmaceuticals (India). Glass distilled water was prepared in the laboratory.

### Preparation of standard stock solution:

Standard stock solution containing AM and CE was prepared by dissolving 10 mg of AM and CE separately in 20 ml of methanol and then final volume of both the solutions was made up to 100 ml with glass distilled water to get stock solution containing 100 μg/ml of each AM and CE in two different 100 ml volumetric flasks.

### Determination of absorptivity:

By appropriate dilution of standard drug solutions with glass distilled water, six working standard solutions containing 10, 15, 20, 25, 30 and 35 μg/ml of AM and CE were prepared separately and scanned in the range of 200-350 nm. The absorbance were recorded at the selected wavelengths and the absorptivity and molar absorptivity values were determined for AM and CE. Molar absorptivity values determined for AM at 236 and 243 nm were 8455.76 and 10370.8 cm^−1^ mol^−1^ l^−1^, while molar absorptivity values determined for CE at 236 and 243 nm were 9951.63 and 2217.92 cm^−1^ mol^−1^ l^−1^.

### Preparation of mixed standard solutions:

Each marketed tablet formulation of the two drug contains AM 60 mg and CE 5 mg. The standard stock solution of AM and CE was used to prepare seven mixed standards containing 30-48 μg/ml of AM and 7.5-12 μg/ml of CE.

### Framing Equations:

From the molar absorptivity values determined for AM and CE the simultaneous equation is derived for determination of AM and CE in pure drug mixed standards and in its pharmaceutical formulation. The equations framed are C_1_ = [(Q_0_ –Q_2_)/(Q1-Q2)]×[(A)/(a_1_)]-----(1) and C_2_ = [(Q_0_ –Q_1_)/(Q2-Q1)]x[(A)/(a_2_)]-----(2), where C_1_ is concentration of AM in g dm^−3^, C_2_ is concentration of CE in g dm^−3^, a_1_ is absorptivity of AM at 236 nm, a_2_ is absorptivity of CE at 236 nm, Q_0_ is ratio of absorbance of sample at 243 nm and 236 nm, Q_1_ is ratio of absorptivity of AM at 243 nm and 236 nm, Q_2_ is ratio of absorptivity of CE at 243 nm and 236 nm and A is absorbance of sample at isoabsorptive point.

### Sample preparation:

Marketed tablet formulations containing 60 mg of AM and 5 mg of CE were analyzed by this method. From the triturate of 20 tablets, an amount equivalent to 30 mg of AM and 2.5 mg of CE was weighed and transferred to 100 ml volumetric flask. A 5 mg of pure CE was added to the volumetric flask. The contents of the flask were dissolved in the 60 ml of the solvent with the aid of ultrasonication for 10 min. The solution was filtered through whatmann filter paper no. 41 and then final volume of the solution was made up to 100 ml with glass double distilled water to get a stock solution containing 300 μg/ml of AM and 75 μg/ml of CE. After appropriate dilutions, the absorbances were measured and the concentration of each analyte was determined with the equations generated. The statistical data obtained after replicate determinations (n = 9) is shown in Table 2.

## RESULTS AND DISCUSSION

The primary target in developing this liquid chromatographic method was to achieve simultaneous estimation of AM and CE in the pharmaceutical formulation under common conditions that are applicable for routine quality control of this product in laboratories. In RP-HPLC method, mobile phase containing of a mixture of methanol-acetonitrile-water (40:40:20) was selected which produces satisfactory resolution, reasonable retention and acceptable peak shape for both the drugs. The optimization of wavelength was done at different wavelengths for detection by UV detector. In the present investigation, drug solutions of 10 μg/ml of AM and 10 μg/ml of CE were prepared in methanol. AM and CE showed wavelength maxima at 243 nm and 229 nm, respectively. After observing UV spectra of both the drugs, wavelength of 229 nm was selected for further study. Keeping in view the physicochemical properties of both the drugs in the formulation, few drug molecules were tested for use as an internal standard with respect to resolution suitability. HT was found to give good resolution and accurate and precise quantitative results. A flow rate of 1 ml/min resulted in optimum retention times with good resolution and with all the drug components and internal standards eluting within 15 min.

The tablet matrix was also estimated to check interference if any, from the excipients used in the tablet matrix. No significant peaks from tablet matrix were observed in the developed chromatograms indicated there is no interference from the excipients of the tablet matrix. The retention time is 2.792 min for HT, 4.155 min for CE and 10.495 min for AM, respectively. The run time is less than 15 min.

**Fig. 3:**
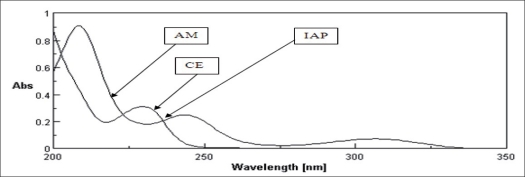
Overlain spectra of AM, CE and isoabsorptive point at 236 nm Overlain spectra of ambroxol hydrochloride (AM), cetirizine hydrochloride (CE) and Isoabsorptive point at 236 nm (IAP).

In absorbance ratio method, methanol: water (1:4) was selected as common solvent. Drug solutions of concentration 10 μg/ml of AM and CE separately were prepared by appropriate dilution of stock solutions. These drug solutions were scanned in the range of 200 to 350 nm to determine wavelength of maximum absorption and isoabsorptive point. AM shows characteristic peaks at 207, 243 and 306 nm while absorbance maxima for CE is 229 nm, respectively. The isoabsorptive point was obtained at 236 nm. The wavelengths selected were 243 nm (λ_max_ of AM) and 236 nm (isoabsorptive point). The overlain spectra of AM and CE is shown in fig. 3.

The linearity of responses of the method I for both drugs was verified at six concentration levels 120 to 480 μg/ml for AM and 10-35 μg/ml for CE, respectively. The calibration curve was constructed by plotting response against concentration of the drug. The results showed that an excellent correlation existed between peak area and concentration of each drug within the concentration range tested by this method. The quantitation limit of an individual analyte is the lowest concentration of analyte in a sample, which can be established at a signal to noise ratio of 10. The LOQ of AM and CE was found to be 0.1076 and 0.2160 μg/ml, respectively. The detection limit of an individual analyte is the lowest amount of analyte in a sample, which can be detected but not necessarily quantitated as an exact value. The LOD of AM and CE was found to be 0.0328 and 0.0648 μg/ml, respectively. The data for LOD and LOQ is given in Table 1.

Accuracy was determined by applying developed method to synthetic mixtures of excipients to which known amounts of each drug corresponding to 50, 100 and 150 percent of label claim had been added. The accuracy was then calculated as the percentage of analyte recovered from the tablet matrix. Mean recovery (mean percentage±standard deviation) values were 101.26±1.18 and 99.14±0.25 for AM and CE, respectively. The data for accuracy is given in Table 3.

**TABLE 3:** RECOVERY STUDIES

Method	Label claim (mg/tablet)	Amount added (%)	Total amount added (mg)	concentration recovered* (mg)±SD	% Recovery ±SD	% RSD
Method I	AM 60	50	90	90.42	100.46±0.93	0.92
		100	120	120.89	100.74±0.55	0.56
		150	150	149.23	99.48±0.89	0.88
	CE5	50	7.5	7.61	101.46±0.62	0.63
		100	10	10.11	101.1±0.95	0.96
		150	12.5	12.69	101.52±1.03	1.04
Method II	AM 60	50	90	90.79	100.87±1.02	1.01
		100	120	119.20	99.33±1.11	1.12
		150	150	148.57	99.04±0.82	0.83
	CE5	50	7.5	7.40	98.66±1.06	1.06
		100	10	10.09	100.90±1.10	1.09
		150	12 5	12.74	101.92±0.76	0.77

* indicates that each value is a mean±standard deviation of three determinations;%RSD = Relative standard deviation

The linearity of responses of the method II for both the drugs was verified by preparing different concentration levels 1 to 50 μg/ml and 2 to 80 μg/ml for AM and CE, respectively. Beer's law was obeyed in the concentration range of 5 to 50 μg/ml and 7.5 to 75 μg/ml for AM and CE, respectively. The LOQ of AM and CE were found to be 3.75 and 1.45 μg/ml, respectively. The LOD of AM and CE was found to be 1.12 and 0.45 μg/ml, respectively. The data for LOD and LOQ is given in Table 1. The accuracy was calculated as the percentage of analyte recovered from the tablet matrix. Mean recoveries (mean percentage±standard deviation) values were 99.82±0.62 and 100.35±1.26 for AM and CE, respectively. Results of recovery study are close to the 100 percentage indicating non-interference of common excipients used in the tablet formulation. The data for recovery studies and precision is given in Tables 3 and 4, respectively.

**TABLE 4:** PRECISION DATA

Method	Label claim (mg/tablet)	Concentration μg/ml	Intra-day precision % concentration* ±% RSD	Inet-day precision % concentration ±% RSD
Method I	AM 60	10	100.41 ±0.68	100.62±1.14
		20	99.32±0.99	100.87±1.05
		30	100.13±0.72	100.45±1.13
	CE5	06	99.31±1.06	98.89±0.87
		12	102.19±0.47	101.87±0.41
		18	99.58±1.02	100.29±1.05
Method II	AM 60	40	99.78±1.98	99.01±1.12
		80	100.01 ±0.84	100.10±1.58
		120	101.27±1.25	101.00±1.57
	CE5	25	99.72±0.80	100.91±0.90
		50	100.93±1.06	100.85±1.18
		75	100.53±0.92	98.97±0.71

* Average of three determinations; % RSD = Relative standard deviation

The results of analysis of methods developed were compared with the reported HPTLC method by performing two way anova studies. Anova studies were performed by using software Graphpad Prism 5.0. F test value at confidence interval 99% was found to be less than table F value. Since the F test values is less than the table F value, the difference in the results of analysis between the developed methods is not significant. Results of analysis of anova studies are given in Table 5.

**TABLE 5:** RESULTS OF ANOVA ANALYSIS

Source of variation	Degree of freedom	Sum of squares	Mean square	F
Interaction	2	1.095	0.5475	1.064
Column	2	0.2740	0.1370	0.2662
Row Factor	1	9.783	9.783	19.01
Residual	12	6.176	0.5146	--
Total	17	17.328	10.9821	--

AM and CE was simultaneously determined in tablet matrix using two different analytical methods. The methods developed are simple, accurate, rapid, sensitive and specific. RP-HPLC and absorbance ratio spectrophotometry may be recommended for routine and quality control analysis of investigated drugs in two component pharmaceutical preparations.
